# Characterisation of Ferritin–Lymphocyte Ratio in COVID-19

**DOI:** 10.3390/biomedicines11102819

**Published:** 2023-10-18

**Authors:** Alexander Liu, Robert Hammond, Kenneth Chan, Chukwugozie Chukwuenweniwe, Rebecca Johnson, Duaa Khair, Eleanor Duck, Oluwaseun Olubodun, Kristian Barwick, Winston Banya, James Stirrup, Peter D. Donnelly, Juan Carlos Kaski, Anthony R. M. Coates

**Affiliations:** 1 School of Medicine, University of St Andrews, St Andrews KY16 9TF, UK; aql1@st-andrews.ac.uk (A.L.); rjhh@st-andrews.ac.uk (R.H.); pdd21@st-andrews.ac.uk (P.D.D.); 2 Royal Berkshire NHS Foundation Trust, Reading RG1 5AN, UK; kennethchan1@nhs.net (K.C.); dr.chuk@doctors.org.uk (C.C.); beckyljohnson@live.co.uk (R.J.); duaa_khair@hotmail.com (D.K.); eleanor.duck@gmail.com (E.D.); seun.r.olubodun@gmail.com (O.O.); kristianbarwick@outlook.com (K.B.); jimstirrup@hotmail.com (J.S.); 3 Royal Brompton Hospital, London SW3 6NP, UK; w.banya@rbht.nhs.uk; 4 Molecular and Clinical Sciences Research Institute, St George’s University of London, London SW17 0QT, UK; jkaski@sgul.ac.uk; 5 Institute of Infection and Immunity, St George’s University of London, London SW17 0QT, UK

**Keywords:** coronavirus disease 19, ferritin–lymphocyte ratio, inflammatory biomarkers, risk stratification, C-reactive protein, white cell count

## Abstract

**Introduction:** The ferritin–lymphocyte ratio (FLR) is a novel inflammatory biomarker for the assessment of acute COVID-19 patients. However, the prognostic value of FLR for predicting adverse clinical outcomes in COVID-19 remains unclear, which hinders its clinical translation. **Methods:** We characterised the prognostic value of FLR in COVID-19 patients, as compared to established inflammatory markers. **Results:** In 217 study patients (69 years [IQR: 55–82]; 60% males), FLR was weakly correlated with CRP (R = 0.108, *p* = 0.115) and white cell count (R = −0.144; *p* = 0.034). On ROC analysis, an FLR cut-off of 286 achieved a sensitivity of 86% and a specificity of 30% for predicting inpatient mortality (AUC 0.60, 95% CI: 0.53–0.67). The negative predictive values of FLR for ruling out mortality, non-invasive ventilation requirement and critical illness (intubation and/or ICU admission) were 86%, 85% and 93%, respectively. FLR performed similarly to CRP (AUC 0.60 vs. 0.64; *p* = 0.375) for predicting mortality, but worse than CRP for predicting non-fatal outcomes (all *p* < 0.05). On Kaplan–Meier analysis, COVID-19 patients with FLR values > 286 had worse inpatient survival than patients with FLR ≤ 286, *p* = 0.041. **Conclusions:** FLR has prognostic value in COVID-19 patients, and appears unrelated to other inflammatory markers such as CRP and WCC. FLR exhibits high sensitivity and negative predictive values for adverse clinical outcomes in COVID-19, and may be a good “rule-out” test. Further work is needed to improve the sensitivity of FLR and validate its role in prospective studies for guiding clinical management.

## 1. Introduction

In patients with acute coronavirus-19 (COVID-19), clinical risk stratification is important for guiding management decisions [1]. The development of novel inflammatory markers that can inform about prognosis in patients presenting to the emergency department is highly desirable [1]. Recent interest has emerged on the use of combination inflammatory biomarkers for the assessment of prognosis in patients with COVID-19 [2,3,4,5]. One such biomarker is based on the combination of serum ferritin and lymphocyte assessments, the ferritin–lymphocyte ratio (FLR), which has shown some early promise for assessing the prognosis of patients with COVID-19 [6].

Serum ferritin is an inflammatory marker that increases in response to systemic infection and exhibits both host-protective and immuno-modulatory functions [7]. Whilst elevated ferritin levels have been linked to adverse outcomes in acute COVID-19 patients [8], conflicting reports have also emerged which cast doubts on its ability to accurately predict mortality [9]. Much like other routine inflammatory markers, the major drawback of ferritin as a biomarker is that it is a non-specific acute-phase reactant [10], rather than a specific indicator of the severity of viral infections. Therefore, improving the prognostic value of ferritin in viral infections may help to improve its clinical applications in COVID-19.

Lymphopenia is commonly observed in acute COVID-19 [11]. Lymphocyte counts are routinely measured in clinical practice [11] and when combined with ferritin, could potentially redirect its prognostic value towards becoming a more viral-specific biomarker. As a novel inflammatory index [6], FLR can be calculated using routinely available blood tests [6]. The only initial report suggested that FLR levels are related to COVID-19 disease severity and can accurately predict mortality [6]. In a single-centre retrospective study based on 331 patients, Aygun et al. reported that the ratio of ferritin and lymphocyte percentage achieved an area under the curve of 0.909 for predicting mortality in COVID-19 patients [6].

Despite early promise, the biomarker characteristics and prognostic value of FLR in relation to established inflammatory markers, such as C-reactive protein (CRP) and white cell count (WCC), remain unknown. Furthermore, the prognostic value of FLR has not been re-assessed in another centre or a different COVID-19 patient population, meaning that the potential expansive usefulness of FLR remains largely unknown. These unknown factors significantly limit the clinical applicability of this novel biomarker, and require elucidation.

In this study, we set out to characterise FLR as a biomarker in terms of the following: (i) its distribution within an acute COVID-19 population; (ii) its correlation with other inflammatory markers; and (iii) its predictive value for adverse clinical outcomes as compared to established markers of infection. We also sought to further assess the prognostic value of FLR in a different COVID-19 population to the previous report, to assess the replicability of this biomarker. We hypothesised that FLR has good prognostic value in acute COVID-19 patients.

## 2. Materials and Methods

### 2.1. Study Subjects

The study population included patients admitted to a UK general hospital (Royal Berkshire NHS Foundation Trust, UK) between 3 February 2020 and 9 May 2020. Patients were included if they (i) were 18 years or older, (ii) had been diagnosed with acute COVID-19 using real-time reverse transcriptase polymerase chain reaction (PCR) testing of nasopharyngeal swabs, or (iii) had serum ferritin and lymphocyte assessment on admission to hospital. Patients were excluded if they did not undergo assessment of other inflammatory markers such as CRP on admission (*n* = 1); were transferred to another hospital during their admission and lost to follow-up (*n* = 1); had been treated at another hospital prior to admission (*n* = 2); or had ferritin assessed later than 24 h post admission (*n* = 2). A final total of 217 patients were included in the study.

### 2.2. Data Collection

Clinical data and laboratory test results were collated according to a standardised protocol by a team of COVID-19 pandemic frontline clinicians. To achieve familiarity with data collection procedures, each observer first collected ten sample cases, which were validated for accuracy against the medical records by an independent observer. Upon satisfactory completion of the sample collection process, the observers then completed the data collection. To further ensure accuracy, after all data were collected, samples of data were independently validated again by two observers against the medical records.

### 2.3. Ethical Approval

This study was given COVID-19 Fast-Track Approval by the Health Research Authority (HRA) and Health and Care Research Wales (HCRW), UK.

### 2.4. Study Endpoints

The primary endpoint was inpatient mortality related to acute COVID-19. The secondary endpoints were (i) requirement for non-invasive ventilation (NIV) related to acute COVID-19, and (ii) critical illness, as defined by a composite of requirement for intubation, mechanical ventilation, or intensive care unit (ICU) admission related to acute COVID-19. FLR was calculated as ferritin (ng/mL) ÷ lymphocyte count (×10^9^/L), as per conventional units [12,13].

### 2.5. Statistical Analysis

Data were checked for normality using the Kolmogorov–Smirnov test [14]. Data for diastolic blood pressures and haemoglobin values were parametric and were expressed as mean (SD) [15]. The remaining data were non-parametric and were expressed as median (inter-quartile range; [IQR]) [15]. Parametric continuous variables were compared using the unpaired Student’s *t*-test [15]. Non-parametric continuous variables were compared using the Mann–Whitney test [15]. Categorical variables were compared using the Chi-square test [16]. Correlations between data groups were assessed using the Pearson’s correlation co-efficient [17]. The diagnostic performance of variables for predicting clinical outcome endpoints was assessed using receiver operator characteristics (ROC) analysis [18], with the optimal (Youden) cutoff displayed as appropriate [19,20]. Inpatient survival was assessed using Kaplan–Meier curves and the logrank test [21]. *p* values <0.05 were considered statistically significant. Statistical analysis was performed by the first author (MedCalc, Version 20.104, Ostend, Belgium) and independently validated by a medical statistician (Stata; Basic Edition version 17.0, Statacorp LLC, College Station, TX, USA).

## 3. Results

### 3.1. Baseline Patient Characteristics

The 217 study patients had a median age of 69 years [IQR: 55–82] and 60% of the patients were males (Table 1). Patients who were non-survivors were older, and had a similar level of symptom burden compared to patients who were survivors of COVID-19 (Table 1). Non-survivors had a higher prevalence of ischaemic heart disease, heart failure and chronic obstructive airways disease than survivors (Table 1).

### 3.2. Clinical and Laboratory Results

Non-survivors had higher FLR values (*p* = 0.026), higher CRP levels (*p* = 0.001) and lower lymphocyte counts (*p* = 0.015) compared to survivors (Table 2). Although the NIV requirement was higher in non-survivors than survivors, both patient groups had similar prevalence of critical illness (Table 2).

### 3.3. FLR Distributions and Correlations

**Figure 1:**
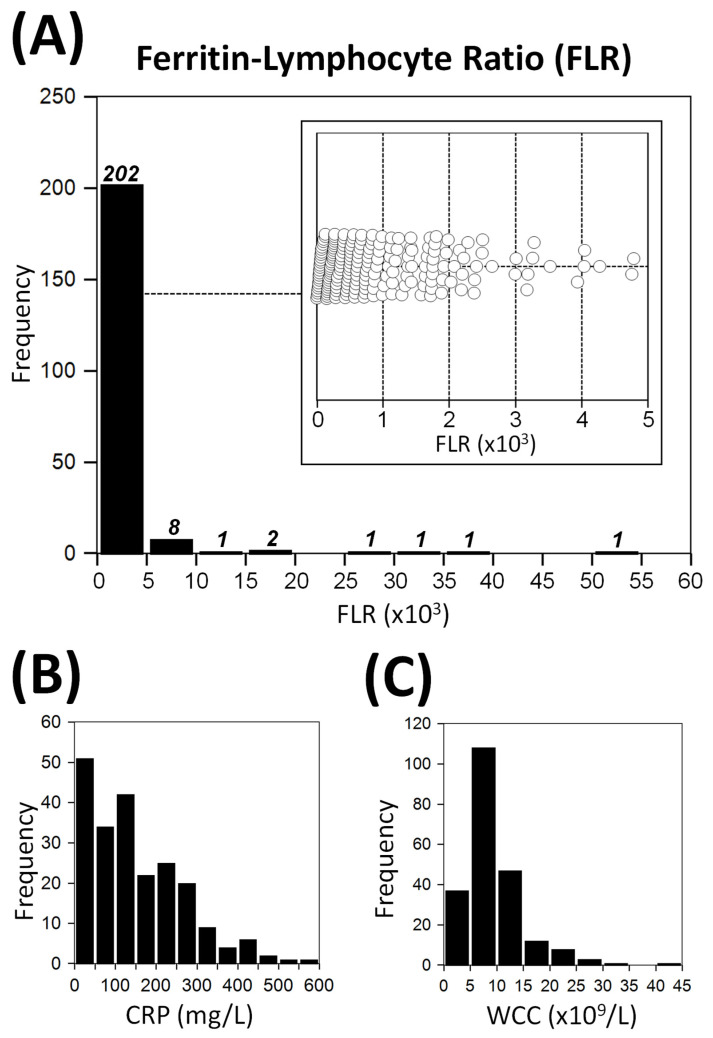
Distribution of ferritin–lymphocyte (FLR) and other inflammatory markers. Panel (**A**): the numbers of patients in each FLR range are indicated above the bars; the inset shows distribution of FLR values between 0 and 5000. Panels (**B**,**C**) demonstrate the distribution of C-reactive protein (CRP) and white cell count (WCC) levels, respectively.

Figure 1 shows the distribution of FLR, CRP and WCC in the study patients. The FLR distribution (median 711 [272–1271]) demonstrates a positive skew, with over half of all values (61%) being clustered under 1000, and the majority (93%) of values falling under 5000 (Figure 1).

**Figure 2:**
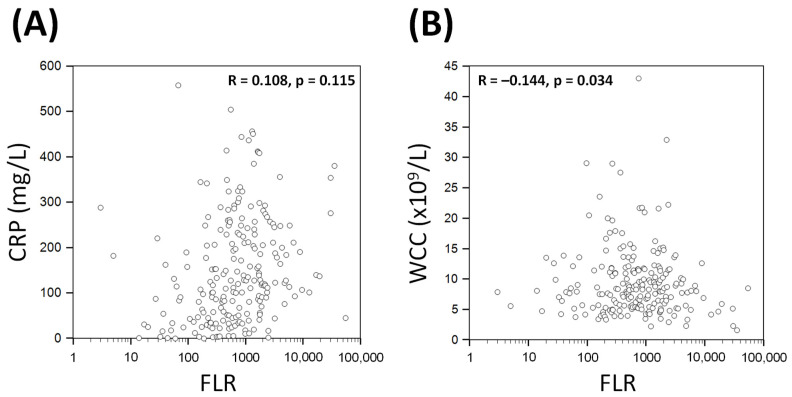
Correlations of ferritin–lymphocyte ratio (FLR) with CRP (Panel (**A**)) and WCC (Panel (**B**)). Each point represents data from a single patient. Pearson’s correlation co-efficient (R) values as indicated. CRP: C-reactive protein; WCC: white cell count.

FLR demonstrated weak correlations with CRP (Pearson R = 0.108, *p* = 0.115) and WCC (R = −0.144; *p* = 0.034, Figure 2)

### 3.4. Diagnostic Performance for Predicting Clinical Outcomes

**Figure 3:**
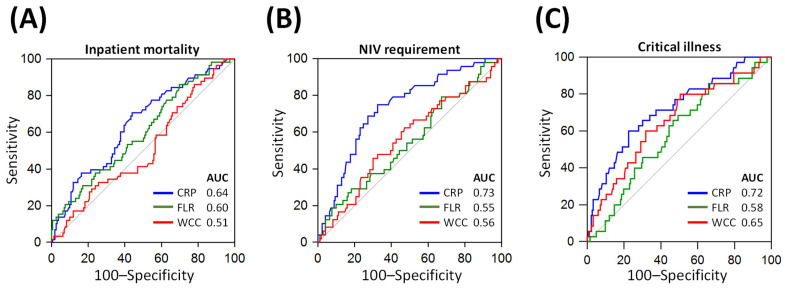
Comparison of the prognostic value of ferritin–lymphocyte ratio (FLR) with other inflammatory markers. Receiver operator characteristics (ROC) curves are shown for predicting inpatient mortality (Panel (**A**)), requirement for non-invasive ventilation (Panel (**B**)) and critical illness, as defined by a composite of intubation, mechanical ventilation or intensive care unit admission (Panel (**C**)). AUC: area under the ROC curve; CRP: C-reactive protein; WCC: white cell count.

For predicting inpatient mortality in acute COVID-19 patients, FLR achieved an AUC of 0.60 (95% CI: 0.53–0.67; Figure 3A) on ROC analysis, with an optimal (Youden; [19]) cut-off of 286 yielding a sensitivity of 86% (95% CI: 75–94%), a specificity of 30% (95% CI: 23–38%), a positive predictive value (PPV) of 31% (95% CI: 28–34%) and a negative predictive value (NPV) of 86% (95% CI: 75–92%). FLR performed similarly to CRP (AUC 0.60 vs. 0.64; *p* = 0.375) and WCC (AUC 0.60 vs. 0.51; *p* = 0.115) for predicting mortality.

For predicting the non-invasive ventilation (NIV) requirement, FLR achieved an AUC of 0.55 (95% CI: 0.48–0.62; Figure 3B) on ROC analysis, with a cut-off of 356 yielding a sensitivity of 79% (95% CI: 65–90%), a specificity of 33% (95% CI: 26–40%), a PPV of 25% (95% CI: 22–29%) and a NPV of 85% (95% CI: 75–91%). CRP outperformed FLR (AUC 0.73 vs. 0.55, *p* < 0.001) and WCC (AUC 0.73 vs. 0.56, *p* = 0.003) for predicting NIV requirement. FLR performed similarly to WCC (AUC 0.55 vs. 0.56, *p* = 0.826) for the same purpose.

For predicting critical illness (a composite of intubation and/or ICU admission), FLR achieved an AUC of 0.58 (95% CI: 0.52–0.65; Figure 3C) on ROC analysis, with a cut-off of 368 yielding a sensitivity of 86% (95% CI: 70–95%), a specificity of 34% (95% CI: 27–41%), a PPV of 20% (95% CI: 17–23%) and a NPV of 93% (95% CI: 84–97%). CRP outperformed FLR (AUC 0.72 vs. 0.58; *p* = 0.037), but not WCC (AUC 0.72 vs. 0.65; *p* = 0.375), for predicting critical illness. FLR performed similarly to WCC (AUC 0.58 vs. 0.65; *p* = 0.328) for the same purpose. The diagnostic performance of FLR for adverse clinical outcomes in acute COVID-19 patients is summarised in Table 3.

### 3.5. Survival Analysis

**Figure 4:**
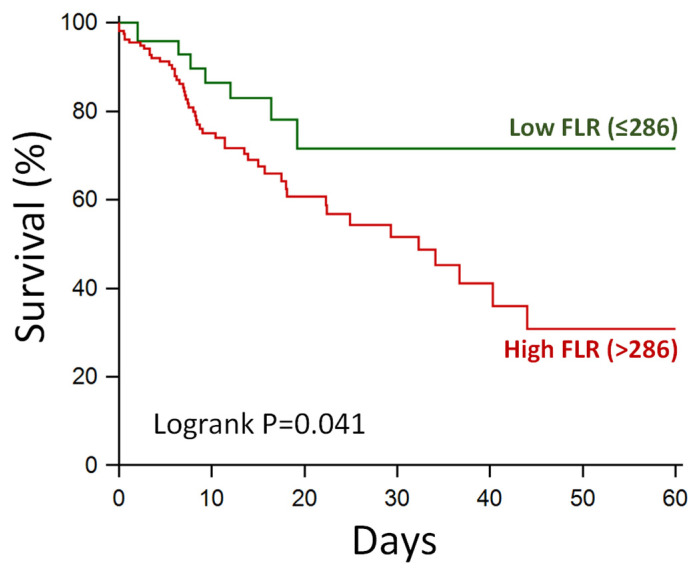
Kaplan–Meier analysis of inpatient survival based on ferritin–lymphocyte ratio (FLR). The optimal FLR threshold (286) was derived from receiver operating characteristics curves.

On Kaplan–Meier analysis, COVID-19 patients with FLR above 286 (ROC cut-off) had worse inpatient survival compared to patients with other FLR levels, *p* = 0.041 (Figure 4).

## 4. Discussion

This study characterised the use of ferritin–lymphocyte ratio (FLR) for assessing prognosis in patients with acute COVID-19, and its relationship with other established inflammatory markers. The main findings are as follows: (i) on a patient group basis, high FLR values appear to be linked to impaired inpatient survival; (ii) on an individual-patient basis, FLR has relatively high sensitivity and negative predictive values (but poor specificity and positive predictive values) for predicting adverse clinical outcomes; (iii) FLR has a positively skewed distribution within the study population; (iv) FLR is weakly correlated with other established inflammatory markers; and (v) FLR performed similarly to CRP for predicting adverse clinical outcomes in acute COVID-19 patients.

The results suggest that FLR may be a good rule-out test for adverse outcomes in COVID-19, similar to other diagnostic tests previously investigated [22,23,24]. Further work is required to validate the potential of FLR for reducing COVID-19 hospitalisation in combination with other inflammatory biomarkers.

### 4.1. FLR as a Rule-Out Test for Adverse Outcomes in COVID-19

Aygun et al. showed in 331 COVID-19 patients that FLR demonstrated high sensitivity for predicting inpatient mortality [6]. The current study goes further, to show that FLR not only had a high sensitivity for predicting mortality, but also for predicting non-fatal clinical outcomes such as NIV requirement, intubation and ICU admissions in COVID-19 patients [6]. On a diagnostic level, the high sensitivity levels of FLR are coupled with high negative predictive values (NPV) for ruling out adverse outcomes, noting the fact that NPV is, in part, prevalence-driven and would likely vary in different clinical settings.

The prognostic value of FLR was further demonstrated in the current study using Kaplan–Meier analysis. Patients with FLR values below the 286 (Youden point) threshold identified on ROC curves had consistent inpatient survival, which was significantly better than patients with FLR above the 286 threshold. This suggests that using this threshold, there may be a scope for FLR to select low-risk (vs. high-risk) patients, which deserves further investigation.

In the study by Aygun et al., FLR achieved an AUC of 0.909 on ROC analysis for predicting inpatient mortality [6], which is higher than the AUC of 0.60 demonstrated in the current study (Figure 3) for the same purpose. Aygun and colleagues also found a higher specificity of FLR for predicting mortality compared to this current study (65.2–82.8% vs. 30%, respectively). The difference in the performance of FLR across the two studies is likely multi-factorial, which could include different study sample sizes, variations in patient demographics, selection criteria used and follow-up strategies. The lack of standardisation for FLR measurements across the two studies can contribute to the differences in the observed results [6]. For instance, lymphocyte assessment was performed as lymphocyte count in this study, and as percentage lymphocyte percentage values in the study by Aygun et al. [6]. A further standardised study to validate FLR in a larger and multi-centred population may provide a better adjudication of the overall prognostic value of FLR.

### 4.2. FLR as a Combination Biomarker

Effective combinations are commonplace in medicine, ranging from clinical risk scores [25,26,27] to multi-drug chemotherapy [28]. Combination biomarkers have recently been investigated for their roles in prognosticating COVID-19 patients [3,5,6,29]. As a combination biomarker, FLR is designed to utilise the prognostic value of both ferritin and lymphopenia in COVID-19, to produce an unique and potentially more viral-specific surrogate indicator. The results of this study demonstrate that FLR is indeed a standalone biomarker that correlates poorly with other inflammatory markers such as CRP and WCC (Figure 2). Moreover, the distribution of FLR was also dissimilar to CRP and WCC, with most values clustered at the extreme low end of the spectrum (Figure 1).

In addition to validating the prognostic value of FLR, the current study puts FLR into further context by comparing it to other established inflammatory markers in widespread clinical use, namely WCC and CRP. Whilst FLR was comparable to WCC for predicting adverse outcomes in COVID-19 overall, FLR underperformed compared to CRP in this regard. The results suggest that FLR may be better used in combination with clinical assessment and other biomarkers rather than as a standalone prognostication tool in COVID-19 patients. The combined use of FLR and CRP for risk-stratifying COVID-19 patients deserves further validation.

### 4.3. Improving FLR

The weakness of FLR appears to lie in its low specificity for adverse outcomes. Although diagnostic cutoffs are arbitrary and changing the FLR threshold would alter the sensitivity and specificity balance, improving the overall specificity of FLR in COVID-19 appears to be an important target of any further work aiming to bring this biomarker into clinical practice.

Improving the specificity of FLR for infections requires the fine-tuning of both ferritin and lymphocyte count as prognostic markers. Ferritin, by nature, is a non-specific acute-phase reactant with multiple mechanisms of action in host–pathogen interactions [7]. It may act to deprive pathogens of iron for growth or provide immune-modulatory and anti-inflammatory functions [7]. Iron is also understood to be vital to many bacteria for the formation of biofilms [30,31]. It remains unclear whether ferritin elevations are a bystander of the systemic inflammation often seen in sepsis or an active player in the pro-inflammatory cascade activation in severe infections [7]. The answers to these important questions may provide the keys to improving the specificity of ferritin as a biomarker of inflammation and prognosis in COVID-19.

Low lymphocyte counts occur not only in acute COVID-19 but also in other viral infections [32]. Therefore, developing a more specific lymphocyte-based biomarker for COVID-19 represents another target for improving the clinical value of FLR. Recent evidence suggests that particular lymphocyte subgroups, CD4+ and CD8+ T-cells, may demonstrate greater specificity for COVID-19 [33]. The expression of certain surface markers such as CD38 and PD-1 on T-cells are also associated with unfavourable clinical outcomes [34]. Further, the exhaustion of lymphocyte function has been observed in COVID-19 patients [34,35], which may lead to reduced viral clearance [35]. Therefore, further biomarker development involving the analysis of lymphocyte subgroups and function, rather than lymphocyte count alone, may offer greater prognostic specificity [33,34,35].

### 4.4. Limitations and Future Directions

This was a retrospective analysis, and larger studies are required to improve the specificity of FLR. Given the high sensitivity and negative predictive values of FLR for predicting adverse clinical outcomes in acute COVID-19 and its ability to stratify patients with preserved and impaired survival, FLR is a combination biomarker that deserves further development. One of the important obstacles to overcome in any biomarker introduction lies in standardising the method of its measurement. Thus far, only two studies (Aygun et al. [6] and this study) have investigated the use of FLR or ferritin lymphocyte percentage ratio (FLPR) in COVID-19 patients, and future studies may also be dictated by local hospital practices in how blood lymphocytes are assessed in the clinical setting. In the hospital where this study was performed, and in most hospitals in the UK, lymphocyte assessments from blood samples are expressed as lymphocyte counts in clinical practice, and this dictated how FLR was measured in this study. Further work is needed to assess the comparability of FLR and FLPR, as well as their relative prognostic values in COVID-19.

Iron overload can be an important confounder to ferritin and FLR in the assessment of COVID-19 severity. Indeed, iron overload is known to be important in the pathogenesis of COVID-19 [36]. Furthermore, the effect of COVID-19 on patients with pre-existing conditions which induce iron overload, such as hemochromatosis and haemoglobinopathies, remain incompletely explored [37,38]. These form important areas of further research. This study could not offer insight into the interaction of ferritin with other more sophisticated inflammatory markers such as interleukins, which may provide further perspectives for prognosticating COVID-19 patients. Future studies investigating the use of other biomarkers for iron status, such as soluble transferrin receptors (sTfRs), can further enrich the prognostic assessment of patients with COVID-19. The study was performed at a time when cortico-steroid therapy was not routinely administered, and vaccination programs were yet to be implemented. Further work is therefore needed to validate FLR for use in these patient groups.

## 5. Conclusions

FLR has prognostic value in COVID-19 patients, and appears unrelated to other inflammatory markers such as CRP and WCC. FLR exhibits high sensitivity and negative predictive values for adverse clinical outcomes in COVID-19 and may be a good “rule-out” test. Further work is needed to improve the sensitivity of FLR and validate its role in prospective studies for guiding clinical management.

## Figures and Tables

**Table 1:** Baseline patient characteristics.

	All Patients (*n* = 217)	Survivors (*n* = 159)	Non-Survivors (*n* = 58)	*p* Value
Age (years)	69 (55–82)	64 (52–80)	75 (66–83)	0.001
Male (%)	130 (60)	95 (60)	35 (60)	0.937
BMI (kg/m^2^)	26 (22–31)	26 (22–31)	26 (23–35)	0.478
Symptoms				
Chest pain	23 (11)	19 (12)	4 (7)	0.285
Cough	123 (57)	92 (58)	31 (53)	0.561
Dyspnoea	123 (57)	86 (54)	37 (64)	0.202
Fatigue	55 (25)	40 (25)	15 (26)	0.916
Fever	112 (52)	87 (55)	25 (43)	0.130
Comorbidities				
Atrial fibrillation	30 (14)	19 (11)	11 (19)	0.185
Ischaemic heart disease	33 (15)	15 (9)	18 (31)	<0.001
Heart failure	27 (12)	15 (9)	12 (21)	0.026
Hypertension	101 (47)	73 (46)	28 (48)	0.757
Diabetes	77 (35)	52 (33)	25 (43)	0.157
Dyslipidaemia	26 (12)	17 (11)	9 (16)	0.341
Current/ex-smoker	66 (32)	44/153 (29)	22/53 (42)	0.086
CKD	74 (34)	49 (31)	25 (43)	0.091
COPD	27 (12)	14 (9)	13 (22)	0.007
Asthma	23 (11)	16 (10)	7 (12)	0.671
CVA/TIA	24 (11)	15 (9)	9 (16)	0.206
Medications				
ACEi/ARB	57 (26)	40 (25)	17 (29)	0.532
Aspirin	40 (18)	26 (16)	14 (24)	0.191
Beta-blockers	49 (23)	29 (18)	20 (34)	0.011
Statins	91 (42)	59 (37)	32 (55)	0.017

ACEi: angiotensin-converting enzyme inhibitor; ARB: angiotensin receptor blocker; BMI: body mass index; CKD: chronic kidney disease; COPD: chronic obstructive pulmonary disease; CVA: cerebrovascular accident; TIA: transient ischaemic attack.

**Table 2:** Patient observations, laboratory results and complications.

	All Patients (*n* = 217)	Survivors (*n* = 159)	Non-Survivors (*n* = 58)	*p* Value
Observations on admission				
Temperature (°C)	37.2 (36.6–37.9)	37.1 (36.6–38.0)	37.2 (36.7–37.9)	0.792
SBP (mmHg)	130 (116–146)	131 (117–147)	127 (111–138)	0.033
DBP (mmHg)	75 ± 14	76 ± 14	71 ± 16	0.014
Respiratory rate (/min)	22 (19–28)	20 (19–26)	23 (20–30)	0.053
Laboratory results				
FLR	711 (272–1722)	662 (250–1543)	848 (447–2157)	0.026
Ferritin (ng/mL)	697 (265–1236)	663 (226–1216)	843 (392–1493)	0.108
Lymphocyte count (×10^9^/L)	0.87 (0.63–1.21)	0.90 (0.64–1.31)	0.73 (0.50–1.04)	0.015
CRP (mg/L)	123 (53–229)	111 (44–208)	159 (101–282)	0.001
Haemoglobin (g/L)	123 ± 24	125 ± 25	118 ± 20	0.027
WCC (10^9^/L)	8.1 (5.5–11.5)	7.9 (5.7–11.6)	8.6 (5.3–11.2)	0.815
Platelet Count (10^9^/L)	234 (183–300)	238 (194–292)	221 (160–314)	0.344
Sodium (mmol/L)	138 (134–140)	137 (134–140)	138 (134–141)	0.635
Potassium (mmol/L)	4.3 (3.9–4.7)	4.3 (3.9–4.6)	4.5 (4.0–5.0)	0.028
Creatinine (μmol/L)	96 (75–185)	91 (72–153)	123 (83–241)	0.055
Complications				
NIV requirement	48 (22)	26 (16)	22 (38)	0.001
ICU admission	35 (16)	24 (15)	11 (19)	0.493
Intubation	18 (8)	13 (8)	5 (9)	1.000

CRP: C-reactive protein; DBP: diastolic blood pressure; FLR: ferritin to lymphocyte ratio; ICU: intensive care unit; NIV: non-invasive ventilation; SBP: systolic blood pressure; WCC: white cell count.

**Table 3:** Diagnostic performance of ferritin–lymphocyte ratio (FLR) for predicting adverse outcomes.

	Mortality	NIV Requirement	Intubation/ICU
AUC	0.60	0.55	0.58
AUC 95% CI	0.53–0.67	0.48–0.62	0.52–0.65
AUC *p*-value	0.023	0.312	0.098
Optimal cut-off (Youden; [19])	286	356	368
Sensitivity (95% CI)	86% (75–94)	79% (65–90)	86% (70–95)
Specificity (95% CI)	30% (23–38)	33% (26–40)	34% (27–41)
Positive LR (95% CI)	1.2 (1.1–1.4)	1.2 (1.0–1.4)	1.3 (1.1–1.5)
Negative LR (95% CI)	0.5 (0.2–0.9)	0.6 (0.4–1.2)	0.4 (0.2–1.0)
PPV (95% CI)	31% (28–34)	25% (22–29)	20% (17–23)
NPV (95% CI)	86% (75–92)	85% (75–91)	93% (84–97)

AUC: area under the receiver operator characteristics (ROC) curve; CI: confidence interval; NIV: non-invasive ventilation; NPV: negative predictive value; PPV: positive predictive value.

## Data Availability

The terms of the ethical approval do not allow the data to be put in a public domain, nor for the open sharing of patient specific clinical data, even when anonymized. Data access requests may be sent to the Health Research Authority (HRA) and Health and Care Research Wales (HCRW) at approvals@hra.nhs.uk.
